# Effects of Polypharmacy in Elderly Diabetic Patients: A Review

**DOI:** 10.7759/cureus.29068

**Published:** 2022-09-12

**Authors:** Sweta Kumari, Shraddha Jain, Sunil Kumar

**Affiliations:** 1 Department of Community Medicine, Jawaharlal Nehru Medical College, Datta Meghe Institute of Medical Sciences, Wardha, IND; 2 Department of Otorhinolaryngology, Jawaharlal Nehru Medical College, Datta Meghe Institute of Medical Sciences, Wardha, IND; 3 Department of Medicine, Jawaharlal Nehru Medical College, Datta Meghe Institute of Medical Sciences, Wardha, IND

**Keywords:** hypertension, neuropathy, dyslipidaemia, diabetic, polypharmacy

## Abstract

Diabetes is a chronic condition brought on by either insufficient insulin production by the pancreas or inefficient insulin utilization by the body or both. A hormone called insulin controls blood sugar. Multiple co-morbidities can arise as a result of the progressive nature of diabetes, necessitating the use of numerous medications. As one or more medications may be used to treat each ailment, the older population with multimorbidity frequently uses many medications, also known as polypharmacy. Due to polypharmacy, harmful medication interactions, and food-drug interactions can occur. Because of the numerous co-morbidities that already exist, there is an increasing tendency of prescribing polypharmacy.

## Introduction and background

The most typical definition of polypharmacy is when a person takes five or more prescriptions per day. According to several research, polypharmacy is the simultaneous long-term use of several distinct drugs by the same person while taking various prescriptions at the same time [1]. Diabetes mellitus is a metabolic illness in which there is an increase in blood glucose levels, which has an immediate as well as long-term impact on several organs. The most prevalent kind of diabetes is type 2 diabetes mellitus, which occurs when there is insulin resistance initially and later the pancreatic beta cells either produce defective insulin or don't produce enough insulin. Diabetes being chronic, affects nerves, blood vessels, heart, and kidneys, and therefore diabetic people suffer from multiple co-morbidities [2]. Globally, around 50% of people suffer from Diabetes mellitus. They also have co-existing hypertension, they are at high risk for developing microvascular and macrovascular diseases. which eventually results in cardiac cell death, congestive heart failure, and coronary artery diseases. Diabetic people are more likely to take multiple medications to tackle these complications, also, as the patient gets older, he or she develops various chronic conditions and uses multiple drugs as a result [3].

According to California research, 41.5% of diabetic patients received at least one anti-hypertensive medication [4]. Elderly people are at risk for developing polypharmacy due to various reasons such as involvement of liver diseases, arterial disease, poor adherence as in psychiatric patients, also because of using herbal medications and over-the-counter drugs they can suffer from adverse drug interactions [5-8]. Diabetic people are at high risk of developing polypharmacy [9,10]. Approximately 40% of diabetic patients have at least three comorbid chronic conditions, according to data from the Medical Expenditure of Panel survey. A cross-sectional study, in Italy, revealed that 57% of diabetic people use five or more five medications [11]. Additionally, the occurrence of polypharmacy is linked to the prescribing cascade, a situation in which unfavorable drug reactions are mistaken for brand-new medical issues, leading to the prescription of brand-new drugs to address such conditions. In addition to its detrimental effects on health, polypharmacy also increases the chance of hospitalization and drug mistake [12]. This review examined the impact of polypharmacy in senior diabetic individuals with a focus on diabetes and sought to understand the disease.

Search Methodology

**Figure 1:**
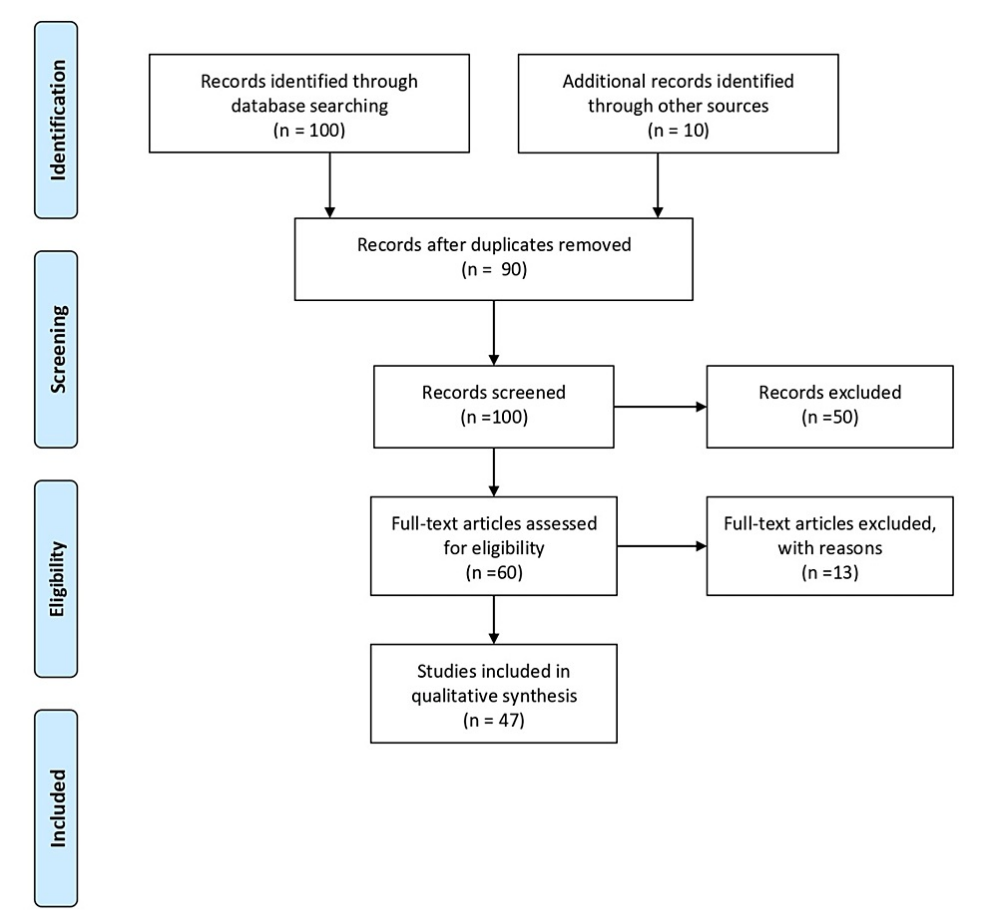
Study Selection Flow Diagram

We conducted extensive research to discover the literature using PubMed and Medline, Cochrane Library, and Web of Science and utilized the following keywords to sensitize or search: Polypharmacy, diabetes mellitus, ageing population, adverse drug interaction, and multiple drug use. All literature was screened for appropriateness by title. We excluded the article which was incomplete, not in the English language, or duplicate. we included the article showing the relation between polypharmacy and Diabetes mellitus. First, we used titles and abstracts to screen the identified studies. A second selection process was applied to the records that were determined to be acceptable at this stage based on the full-text examination. Each time a decision needed to be made, the researchers explored potential differences in the study choice until they came to an agreement. Figure 1 shows the flow of study selection. The following data was collected for each study: Name of the first author, year of publication, research population, age, sex, the definition of polypharmacy, outcome, the prevalence of polypharmacy among people with diabetes], key findings, and conclusion.

## Review

Glycaemic control is the main goal of treating diabetes, and medication is the cornerstone of clinical diabetic care. According to Chelliah et al., intensive care for hyperglycemia is the main problem and obstacle to reaching normal glycaemic targets in older individuals with diabetes. According to Shorr et al., people above the age of 80 and who use five or more concurrent drugs have the highest risk of hypoglycemia. Along with polypharmacy, there is also the fact that at least half of the pharmacologic treatments for type 2 diabetes now in use have hypoglycemia as a serious side effect [13,14]. In order to gauge the incidence of polypharmacy and determine the possibility of drug-drug interactions, which are a side effect of polypharmacy, Ibrahim et al. looked at the medication regimens of diabetic patients receiving home health care services. A total of 139 diabetic individuals made up the sample, with a mean age of 74 and an average of three comorbid conditions. The results of the study showed that 88% of the participants had polypharmacy (> 5 medications) and that the average number of prescribed medications taken each day was 8.9 (SD 3.4), with a range of 2 to 19; 38% of the patients had a severe drug-drug interaction, while 92.8% were at risk for moderate drug-drug interactions, and 70.5% might have mild drug-drug interactions [15].

Sharkey et al. identified the therapeutic prescription medicine categories that these people utilized as well as the factors that contributed to their use of several therapeutic categories. A total of 326 participants, aged 60 and older, from the baseline Nutrition and Function Study in-home evaluation, were used as the source of the data for analysis. According to survey findings, 6.4 (SD 4.2) different prescription medications were taken every day on average, and 3.7 different therapeutic categories were used on average (SD 1.9). The majority of participants-more than 72%-took drugs from three to four different therapeutic categories, and 31.6% utilized drugs from more than five different therapeutic categories. Arthritis and hypertension were the most common concomitant illnesses, each accounting for 78.8% of all cases. Heart issues, including congestive heart failure (63.5%), diabetes (37.4%), and lung disease (37.4%) were the next most common conditions (28.2%) [16]. The study's findings are consistent with the body of literature reporting the increased risk of harmful drug-drug interactions that may change the pharmacokinetic/pharmacodynamic characteristics of medications and how well older adults use drugs.

Yang et al. in their population-based cross-sectional study in Taiwan, including 316 diabetic patients of age more than 60 years found out that polypharmacy was high in elderly diabetic individuals. Polypharmacy was described by them as the concomitant use of more than equal to five drugs and assessed the patients for the quality of life. They found out that polypharmacy was more significant in elderly people, that is 46.6% in their study. The number of drugs had a considerable impact on the social area of quality of life [17]. Al Musawe et al. conducted a cross-sectional study using data from 670 elderly diabetic patients. They examined whether polypharmacy and possibly inappropriate medication use was linked to low quality of life in diabetic patients. Potentially inappropriate medications (PIMs) were identified using STOPP (Screening Tool of Older Persons' Prescriptions) criteria version 2, and quality of life (QoL) was assessed using the three-level Euro Quality of life five-dimensional questionnaire, which includes mobility, self-care, usual activities, pain and discomfort, and anxiety and depressive symptoms. They found out that 72.9% of diabetic people were consuming more than five drugs; 36.11% of the total population had at least one PIMs. The use of many medications has been linked to serious issues with movement, daily activities, personal care, discomfort, anxiety, and depression. Poorer quality of life was linked to polypharmacy in older diabetes patients [18]. Amin et al., in their cross-sectional study including 150 diabetic people of age more than 65 years, found out that polypharmacy is prevalent in older diabetic individuals that are 61%, although they defined polypharmacy as the use of more than equal to four drugs [19].

In a population-based cross-sectional study with 214 nursing home residents, 57 of whom had diabetes, Cracken et al. assessed polypharmacy in relation to outcomes related to diabetes, such as glycaemic management. Polypharmacy was defined as taking more than nine different medications, and it was discovered that 57.9% of people had it. They concluded that polypharmacy is linked to more aggressive diabetes therapy [20]. Oktora et al. performed a cross-sectional study for over five years including 24809 people using prescription databases from University Groningen IADB.nl, in diabetic people of age more than 45 years. They defined polypharmacy as the usage of more than five drugs because 61.5% of the population was over 65. They were evaluated for polypharmacy and the usage of possibly inappropriate medicines. They found that, over the course of their study, older people had an increased incidence of polypharmacy (66.2%) while older adults who had polypharmacy and at least one possibly inappropriate prescription had a declining trend (24.9%) [21].

In their cross-sectional investigation, Noale et al. included 1,342 diabetics older than 65 years. The multidimensional impairment was evaluated. They identified 57.1% of people as having polypharmacy, which they defined as being prescribed at least five medicines simultaneously. Additionally, they found that women were more affected than men and that patients with polypharmacy had longer median durations of diabetes, high BMI, and hypoglycaemic episodes [22]. A study by Forbes et al. that included 35717 diabetic people showed that polypharmacy was linked to greater mortality in diabetic patients and even more so in non-diabetic patients throughout a 10-year prospective analysis involving 60.7% of diabetic patients and 52.3% of non-diabetic patients [23]. Indu et al. demonstrated the relationship between comorbidity and age in diabetic patients in a cross-sectional study. They found that 83.3% of the population had at least one comorbid condition, with hyperlipidemia (70.7%) and hypertension (47.3%) being the most prevalent. The geriatric age group also had the highest prevalence of polypharmacy. As people age, their comorbidities increase along with it, adding to the stress of taking pills. Depending on the prescription, two to nine different medications may be included [24]. In contrast, a similar study in Gujarat, India, showed that males were more likely to have diabetes than women; 50.4% of men had the disease compared to 49.6% of women; the average number of medications prescribed was 5.56 2.52 [25]. According to research conducted in Ahmedabad, India, patients receiving prescriptions for major side effects were typically advised to take five different medications that are antitubercular, antiretroviral, and antimicrobial drugs; they are the most common causal drug groups for serious adverse drugs reactions [26]. 

In a cross-sectional study involving 8932 individuals with diabetes, Alwhaibi et al. found that 77.9% of adults use five or more medications and that polypharmacy was more common in older adults aged more than equal to 60 years than in patients between the ages of 18 and 29. The percentage of polypharmacy among women with diabetes is much greater. Additionally, diabetic patients who had two or more chronic complications compared to those who had none had considerably greater rates of polypharmacy [27]. According to studies, women utilize prescription and over-the-counter drugs and healthcare services at higher rates than males in the general population [28-31]. This might be the case because women are more likely than males to be worried about their health and use healthcare services more frequently [32]. A total of 806 diabetics. between the ages of 59 and 70, were included in a cross-sectional survey conducted in Vietnam. The majority of them were female, and it was determined that 40.8% of them had polypharmacy, which includes dietary supplements in addition to prescription and over-the-counter medications, as opposed to 7.8% who met the criterion of polypharmacy. This demonstrates how many people choose non-pharmaceutical treatments for their illnesses [33]. According to a comparable study from Vietnam, one explanation for this is that some patients think their diabetes treatment will be more effective if they combine conventional, herbal, and traditional therapy [34].

People with type 2 diabetes mellitus even quit taking prescribed medications when using traditional medicine, according to a study conducted among Vietnamese immigrants in the United States, as they were concerned about the side effects of prescribed medications [35]. In Vietnam, traditional and herbal medicine is also regarded as a form of treatment that has few to no adverse effects and cheaper overall expenses [36,37]. In addition, those who experienced diabetes-related distress had a greater odds ratio of polypharmacy than those who did not. The outcomes of studies demonstrate that polypharmacy is generally linked to psychological suffering [38]. People with poorly controlled diabetes may experience greater discomfort as a result of the challenges associated with managing their condition, and as a result, they may experiment with various medications to improve their disease's control [39,40]. Diabetes patients need to be routinely checked by healthcare professionals for possibly inappropriate drugs, adverse drug reactions, and drug-drug interactions. An effective influence on reducing polypharmacy has been demonstrated by comprehensive geriatric assessment. Table 1 shows how to manage polypharmacy on a daily basis. It is necessary to manage these patients using comprehensive geriatric assessment, regular exercise, diet management, deprescription of unnecessary drugs, and tight glycaemic controls [41].

**Table 1:** Management of Polypharmacy

Daily pharmacological review of ongoing drugs
Deprescription of unnecessary drugs
Physical activities with realistic goals
Glycaemic targets according to patients’ health
Healthy dietary habits

It was clear from the above studies that, as age advances, the number of comorbid conditions increases in elderly patients and so drug consumption increases. This review summarises the research literature, identifying findings and recommendations that support the need for a thorough evaluation of medication regimens, at each hospital visit, given the high number of medications taken daily by the elderly population who frequently suffer from multiple morbidities and a higher risk of unfavorable clinical outcomes. Polypharmacy is encouraged by the prevalence of diabetes when combined with multiple morbidities. Additionally, due to age-related physiological changes, diabetic elderly patients are at a higher risk of having their blood sugar levels out of control, which results in higher costs and resource utilization.

## Conclusions

We conclude by highlighting the high frequency of polypharmacy among older adults with diabetes and argue that this condition may have a significant impact on a number of health-related outcomes. Because the disease has so many complications, diabetic patients tend to take more medications, and various other factors like cost of polypharmacy, pill burden, and side effects of drugs all contribute to polypharmacy, which worsens the patient's condition. Clinicians must carefully assess the pharmaceutical profile and work with each patient individually to provide the safest, most effective, and most straightforward medication regimen possible. 
